# Cuproptosis correlates with immunosuppressive tumor microenvironment based on pan-cancer multiomics and single-cell sequencing analysis

**DOI:** 10.1186/s12943-023-01752-8

**Published:** 2023-03-24

**Authors:** Yan Qin, Yanling Liu, Xiaoyun Xiang, Xingqing Long, Zuyuan Chen, Xiaoliang Huang, Jianrong Yang, Wei Li

**Affiliations:** 1 Department of Health Management, The People’s Hospital of Guangxi Zhuang Autonomous Region & Research center of Health Management, Guangxi Academy of Medical Sciences, Nanning, Guangxi 530021 People’s Republic of China; 2 Division of Colorectal & Anal Surgery, Department of Gastrointestinal Surgery, Guangxi Medical University Cancer Hospital, Nanning, People’s Republic of China

**Keywords:** Cuproptosis, Pan-cancer analysis, Immune therapy, Prognostic analysis

## Abstract

Recent studies suggest that cuproptosis, a novel mode of cell death, may be associated with the development of cancer. However, no studies are showing its role in tumorigenesis, progression, and prognosis. In the present study, we comprehensively analyzed the expression difference, gene variation and methylation modification of cuproptosis-related genes (CRGs) in pan-cancer. Then, Single sample gene set enrichment analysis (ssGSEA) was used to calculate individual cuproptosis scores (CS). The association of CS with copy number variation, clinical features, immune-related genes, TMB, MSI, and tumor immune dysfunction and exclusion (TIDE) was comprehensively assessed. Single-cell transcriptome sequencing (scRNA-seq) to analyze the activation of cuproptosis in the tumor microenvironment. Immunohistochemistry (IHC) were used to validate the expression of cuproptosis hub-gene. Our study shows that CRGs were significantly expressed in a variety of tumors, and CDKN2A had the highest mutation frequency (49%) in all tumors. A significant increase in the CS was observed in most cancers and were associated with poor prognosis in the majority of tumors. CS was significantly negatively correlated with tumor microenvironment scores in more than 10 tumors and positively correlated with PD-L1 in 11 tumors, suggesting involvement in tumor immune escape. scRNA-seq suggests that CRG scores significantly increased in the cancer cells. This study opens avenues for further research on the role of cuproptosis in the occurrence and development of cancer and the development of targeted therapies based on cuproptosis.

**Supplementary Information:**

The online version contains supplementary material available at 10.1186/s12943-023-01752-8.

## Introduction

Malignant tumors are one of the leading causes of death in humans [1].With the rapid development of life science research, various basic processes within tumor cells, such as signal transduction, regulation of cell cycle, induction of apoptosis, angiogenesis, and interaction between cells and tumor microenvironment, are gradually being elucidated [2]. On this basis, the application of high-throughput screening, combinatorial chemistry and genetic engineering technologies has accelerated the development of anti-tumor drugs [3]. With the popularization of high-throughput sequencing and the improvement of tumor data sharing platform, pan-cancer research has been paid more and more attention. Pan-cancer studies, which combine and analyze cancers of different organ-origin, can provide a deeper and broader understanding of the characteristics of some common signaling pathways that cause cancer, and allow researchers to focus on a relatively large sample size of data. Larger sample sizes increase the statistical power of the data and make it easier to find cancer-related genomic changes, so pan-tumor studies may help uncover drug targets that have never been identified before. In addition, new tumor classification methods based on understanding the characteristics of common signaling pathways could help some cancer patients get treatment that is more tailored to the individual, thus making it more likely that their disease will be relieved [4].

Cuproptosis, a form of cell death induced by copper ion carriers, was first proposed by Tsvetkov et al [5] It has been shown that the mechanism of cuproptosis might be linked to mitochondria, where the accumulation of copper ions in cells induces oligomerization of mitochondrial lipid acylated protein aggregates with reduced Fe-S cluster protein levels leading to proteotoxic stress [6]. Additionally, it has been shown that copper levels in the serum and tumor tissues are significantly altered in patients with a variety of cancers, like breast invasive carcinoma (BRCA), thyroid carcinoma (THCA), ovarian carcinoma (OV), and lung carcinoma (LC) [7] However, a comprehensive understanding of the role of cuproptosis in tumors is still lacking. Hence, it is crucial to explore for the regulatory functions and molecular mechanisms of CRG on tumors to provide new directions and strategies for the clinical treatment of cancer.

## Findings

### Analysis of expression differences, genetic variation, and drug sensitivity of CRGs

**Fig. 1:**
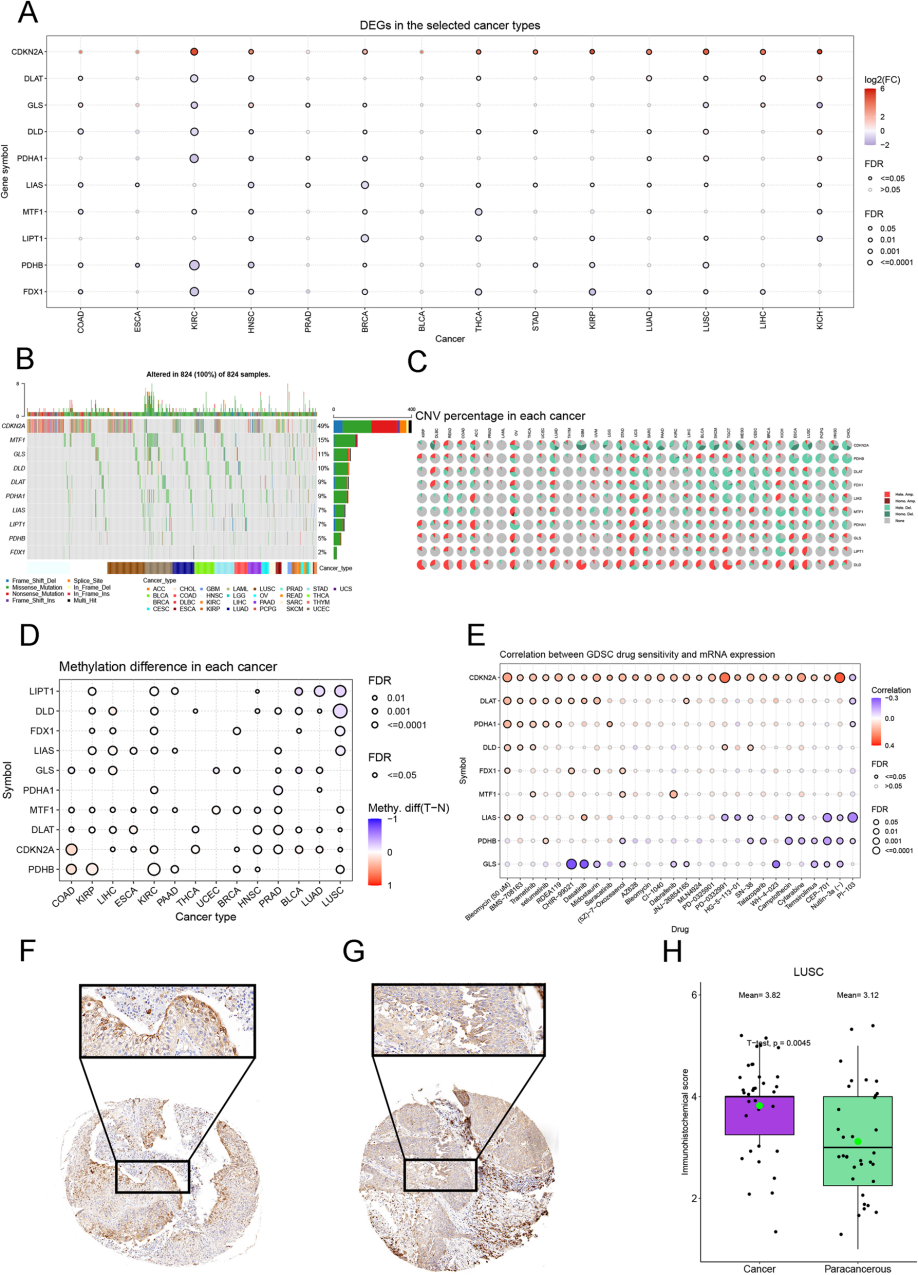
Cuproptosis-related gene expression differences, genetic variation, and drug sensitivity analysis. **A** Differential expression of CRG. The blue dots indicate low gene expression in the tumor and the red dots represent high gene expression in tumors. **B** SNV oncoplot. The cancer map shows the distribution of the classification of SNV types (e.g., missense mutations, frame-shift loss, and nonsense mutations) in CRG. Selected cancer samples are shown together on one side and a bar diagram at the top shows the number of mutations in each sample or gene. **C** CNV pie distribution in 33 cancers. A pie represented the proportion of different types of CNV of one gene in one cancer, and different colors represented different types of CNV. Hete Amp, heterozygous amplification; Hete Del, heterozygous deletion; Homo Amp, homozygous amplification; Homo Del, homozygous deletion; None, no CNV. **D** Methylation difference of CRG in each cancer. The red dots indicate high methylation in cancers, while the blue dots mean low methylation. The solid circles represent FDR < 0.05, a significant correlation. **E** Correlation between GDSC drug sensitivity and mRNA expression. Blue represents a negative correlation, suggesting higher the gene expression, the lower the drug amount required, and the higher the sensitivity. **F**,** G** Immunohistochemical staining results of DLD of lung squamous carcinoma tissue. **H** Statistical results of DLD expression in lung squamous carcinoma and paraneoplastic tissues

Please refer to Supplementary Methods for the materials and methods of this study. The flow chart of the study was shown in Supplementary Fig. 1. Generally, *CDKN2A* was overexpressed in all tumors and significantly highly expressed in *KIRC* (*P* < .05); while *DLAT*, *GLS*, *DLD*, *PDHA1*, *LIAS*, *MTF1*, *LIPT1*, *PDHB,* and *FDX1* were less expressed in almost all tumors, especially in *KIRC* (*P* < .05) (Fig. 1A). The mRNA expression of genes is also regulated by gene variants, and it has been shown that SNPs and CNVs can affect gene expression. We further analyzed the gene variants of CRGs. The cuproptosis-related SNP data were analyzed to observe the frequency of CRGs in each cancer subtype. A total number of 824 tumors revealed that the SNV frequency of CRGs was 100%, suggesting that CRGs mutations are common in tumors. The frequency of CDKN2A mutations was the highest, with 49% of patients having CDKN2A mutations. Missense mutation and nonsense mutation were the main types of CDKN2A mutations (Fig. 1B). SNV percentage analysis revealed a high CDKN2A mutation frequency of 61% in SKCM. (Supplementary Fig. 2). It has been demonstrated that a high frequency of CDKN2A mutations is found in many primary tumors, and that melanoma patients who are carriers of CDKN2A mutations respond better to immunotherapy [8, 9].

The copy number variant (CNV) data of CRG in the TCGA database were analyzed. The CNVs about CRG were mainly heterozygous amplifications and deletions. In most tumors, the expression of CRGs was significantly and positively correlated with CNV amplification (Supplementary Fig. 3). The heterozygous amplification of *DLD* in *KIRP*, *READ*, *ACC*, *GBM*, *SCKM,* and *TGCT* was greater than or equal to 50%. At the same time, in *PDHB* more than 50% heterozygous deletion in *CHOL*, *HNSC*, *LUSC*, *ESCA*, *KIRC*, *USC*, and *UVM* was observed (Fig. 1C). Methylation also affects gene expression. In most tumors, CRGs had a negative correlation between mRNA expression and methylation level, like *LIPT1* in *TGCT* and *PRAD* (*P* < .05) (Supplementary Fig. 4). We compared the methylation levels of CRGs in tumor and non-tumor samples. *LIPT1* methylation was significantly decreased in *BLCA*, *LUAD,* and *LUSC* (Fig. 1D). The drug sensitivity and mRNA expression profile data from *GDSC* were collated to understand the role of cuproptosis in chemotherapy or targeted therapy. Correlation analysis revealed that the *CDKN2A*, *DLAT*, *PDHA1*, *DLD*, and *FDX1* were associated with resistance to trametinib. While *PDHB* and *GLS* were sensitive to various drugs, especially *GLS* for CHIR-99021, dasatinib, and WH-4-023 (Fig. 1E). Thus, it was concluded that aberrant expression of CRGs might mediate sensitivity to targeted drug therapy and chemotherapy. We then found that DLD is the hub gene of CRGs by constructing the PPI network (Supplementary Fig. 5). We performed immunohistochemical experiments on 34 cases of lung cancer with paraneoplastic tissues to further validate the expression of DLD in LUSC and paraneoplastic tissues, and found that DLD was significantly highly expressed in cancer tissues, which was consistent with the results of differential gene expression analysis based on the TCGA database (Fig. 1F-H).

### Prognostic analysis, pathway scores, and immunological characteristics of cuproptosis

**Fig. 2:**
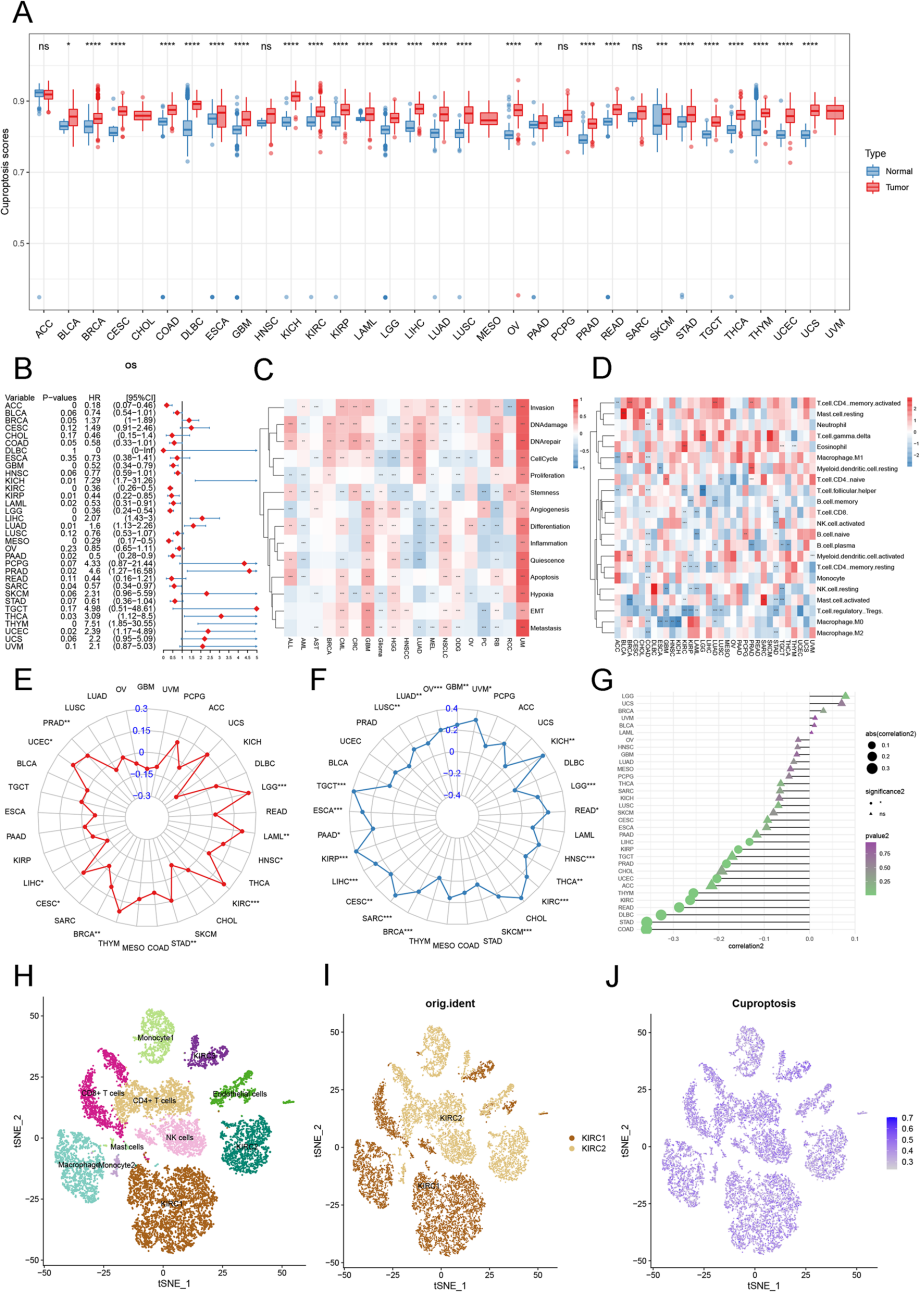
Prognostic analysis, pathway scores, and immunological characteristics of cuproptosis. **A** Combined GTEX and TCGA databases were analyzed for cuproptosis pathway scores. Box lines indicate mean values. **B** OS forest plot for CS. **C** Correlation between CS and cell function. The darker the color, the stronger the correlation. *P < .05, **P < .01, ***P < .001. **D** Correlation analysis of cuproptosis tumor infiltration immune cells. *P < .05, **P < .01, ***P < .001. **E** The correlation of CRG expression and MSI. The circles greater than 0 indicate that cuproptosis gene expression is positively correlated with MSI, but the circles less than 0 mean the opposite. **F** TMB score for cuproptosis. The more distant the points and lines spread outward, the higher the correlation score of the associated tumor is. **G** Correlation of CS with microenvironmental scores. Circles indicate the statistical significance and triangles indicate no statistical significance. Correlation is proportional to the absolute value of the score. **H** tSEN plot representation of KIRC samples with 11 distinct cell types. **I** tSEN plot representation of KIRC from two different samples. **J** Comparison of CSs in different KIRC tumor microenvironment cells. The blue horizontal line on the violin plot indicates the median CS

We constructed the cuproptosis score (CS) using ssGSEA, and the CS was significantly positively correlated with the expression of most of the CRGs (Supplementary Fig. 6), suggesting that the CS can indicate cuproptosis status. We analyzed the CS of normal versus tumor tissues and found that the CS was significantly higher in 26 tumor tissues than in normal tissues, such as BRCA, CESC, and COAD (Fig. 2A). Additionally, the prognostic value of CS in terms of OS performance was assessed. CS was found to be significantly associated with poor prognosis in *LIHC* (*P =* 0), *LUAD* (*P =* .01), *PRAD* (*P = .*02), *THCA* (*P =* .03), *THYM* (*P =* 0), and *UCEC* (*P = .*02) (Fig. 2B). The survival curves for patient OS are shown in Supplementary Fig. 7. The prognostic value of DSS on CRG was evaluated, and it was found that DSS was significantly associated with poor prognosis of *LIHC* (*P =* 0), *SKCM* (*P =* 0), and *UCEC* (*P = .*01) (Supplementary Fig. 8). The survival curves for patient DSS are shown in Supplementary Fig. 9. Similarly, it was observed from the PFI analysis that high expression of cuproptosis was significantly associated with the worsening of PFI in 9 out of 33 tumors (Supplementary Fig. 10). The survival curves for patient PFI are shown in Supplementary Fig. 11. CS could be a potential prognostic marker in specific tumor. In most tumors, CSs were observed to be associated with invasion, DNA damage, DNA repair, in particular uterine myoma (UM) (Fig. 2C). It showed that cuproptosis plays a crucial function in the occurrence, infiltration, diffusion, and metastasis of cancer.

To investigate the relationship between cuproptosis and tumor immune cells, the abundance of 22 different types of immune cells was calculated and it was observed that there was a significant positive correlation of CS with mast cell resting, eosinophil, T cell CD4 memory activated, and myeloid dendritic cell resting. Additionally, it was noticed that there was a significant negative correlation of CS with T cell regulatory Tregs, NK cell resting, Macrophage M0, T cell follicular helper, B cell memory, and B cell plasma (Fig. 2D). There is evidence that copper reduction can inhibit PD-L1 expression through downregulation of the JAK/STAT cell pathway, thereby reducing the negative regulatory effect of tumor immune escape on T cells [10]. We further investigated the correlation of CS with common immunotherapeutic markers. A significant positive correlation of CS with MSI was observed in *LGG*, and *KIRC* (*P* < 0.001) (Fig. 2E). And, CS was significantly positively correlated with TMB in 20 tumors (Fig. 2F). We further analyzed the correlation between CS and tumor microenvironment score, and the results showed that CS was significantly negatively correlated with tumor microenvironment score in more than 10 tumors (Fig. 2G). The above results suggest that CS is closely correlated with tumor immunotherapeutic markers and has the potential to be used as a novel immunotherapeutic marker. In addition, we analyzed the correlation between CS and immune score and stromal score, and the results showed that CS was negatively correlated with immune score in 5 tumors and significantly negatively correlated with stromal score in 20 tumors. The above results suggest that CS is closely related to the tumor microenvironment (Supplementary Fig. 12A, B). Additionally, a multifaceted correlation analysis of cuproptosis was performed that included checkpoints, immune activation genes, immune suppression genes, chemokines and chemokine receptors (Supplementary Fig. 13A-E). Correlation studies between CS and checkpoints showed that there was a significant negative correlation of cuproptosis expression with *TNFRSF14* and *TNFRSF25* in most tumors. *TNFRSF14* and *TNFRSF25* are members of the tumor necrosis factor receptor (TNFR) superfamily [11]. Previous studies have shown that low expression of *TNFRSF14* is associated with a poor prognosis of bladder cancer. *TNFRSF14* may play a role through overexpression in T cells, and its overexpression in T24 cells can stimulate apoptosis and inhibit cell proliferation [12]. Thus, cuproptosis may affect the expression of immune checkpoints and regulate tumor immune escape.

Additionally, scRNA-seq from two *KIRC* samples was analyzed. After QC was conducted using Seurat, 13,124 high-quality single-cell transcriptome information was used for subsequent analyses. Cell clustering analysis based on the tSNE algorithm revealed that the above cells could be classified into 11 clusters, namely KIRC1, KIRC2, KIRC3, Monocyte1, Monocyte2, macrophage, CD4+ T cells, CD8+ T cells, mast cells, NK cells, and endothelial cells (Fig. 2H, I). The expression of different clusters of marker genes is shown in (Supplementary Fig. 14). Investigating, we found significant differences in the abundance of CS in the 11 clusters mentioned above (Supplementary Fig. 15). Further, we used ssGSEA to impute CS for *KIRC* tumor microenvironment cells and compared the differences in CS across cell types (Fig. 2J). The highest CS were discovered in tumor cells, indicating that tumor cells might be more sensitive to target cuproptosis.

## Conclusion

Our study comprehensive evaluation of the role of cuproptosis in pan-cancer. The findings of the study infer that cuproptosis is involved in a majority of mechanisms of tumorigenesis and metastasis and it complicated in the tumor immune escape. The comprehensive assessment of cuproptosis contributes toward improving our understanding of tumorigenesis and remodeling of tumor microenvironment and provides a basis for further studies on the relevance of cuproptosis to tumors.

## Supplementary Information

**Additional file 1: Supplementary Fig. 1.** The flow chart of the study. **Supplementary Fig. 2.** The SNV frequency of CRGs in cancers. **Supplementary Fig. 3.** CNV correlation with mRNA expression. **Supplementary Fig. 4.** Correlation between methylation and mRNA expression. **Supplementary Fig. 5.** Identification of hub genes of CRGs. **Supplementary Fig. 6.** Correlation of cuproptosis multiple genes. **Supplementary Fig. 7.** Kaplan-Meier analysis of the association between CRGs expression and OS. **Supplementary Fig. 8.** The results of cuproptosis for disease-specific survival (DSS) in pan-cancer. **Supplementary Fig. 9.** Kaplan-Meier analysis of the association between CRGs expression and DSS. **Supplementary Fig. 10.** PFI forest plot for CS. **Supplementary Fig. 11.** Kaplan-Meier analysis of the association between CRGs expression and PFI. **Supplementary Fig. 12.** The relationship between CS and immune scores and stroma scores. **Supplementary Fig. 13.** Relationship between CS and immune regulator gene. **Supplementary Fig. 14.** tSEN plot representation of cuproptosis scores in different cell types. **Supplementary Fig. 15.** Comparison of CS of different KIRC tumor microenvironments.

## Data Availability

The data supporting the findings of this study are deposited in the TCGA and the GEO databases. The single-cell sequencing datasets can be found in the online repositories of GEO (GSE152938).
