# Supplementary material for: Cuproptosis correlates with immunosuppressive tumor microenvironment based on pan-cancer multiomics and single-cell sequencing analysis
Source: Mol Cancer. 2023 Mar 24;22:59. doi: 10.1186/s12943-023-01752-8 (PMC10037895; doi:10.1186/s12943-023-01752-8)
Supplement: Supplementary file 1 — Additional file 1: Supplementary Fig. 1. The flow chart of the study. Supplementary Fig. 2. The SNV frequency of CRGs in cancers. Supplementary Fig. 3. CNV correlation with mRNA expression. Supplementary Fig. 4. Correlation between methylation and mRNA expression. Supplementary Fig. 5. Identification of hub genes of CRGs. Supplementary Fig. 6. Correlation of cuproptosis multiple genes. Supplementary Fig. 7. Kaplan-Meier analysis of the association between CRGs expression and OS. Supplementary Fig. 8. The results of cuproptosis for disease-specific survival (DSS) in pan-cancer. Supplementary Fig. 9. Kaplan-Meier analysis of the association between CRGs expression and DSS. Supplementary Fig. 10. PFI forest plot for CS. Supplementary Fig. 11. Kaplan-Meier analysis of the association between CRGs expression and PFI. Supplementary Fig. 12. The relationship between CS and immune scores and stroma scores. Supplementary Fig. 13. Relationship between CS and immune regulator gene. Supplementary Fig. 14. tSEN plot representation of cuproptosis scores in different cell types. Supplementary Fig. 15. Comparison of CS of different KIRC tumor microenvironments. [file 12943_2023_1752_MOESM1_ESM.docx]

**Cuproptosis Correlates with Immunosuppressive Tumor Microenvironment based on Pan-Cancer Multiomics and Single-cell Sequencing Analysis**


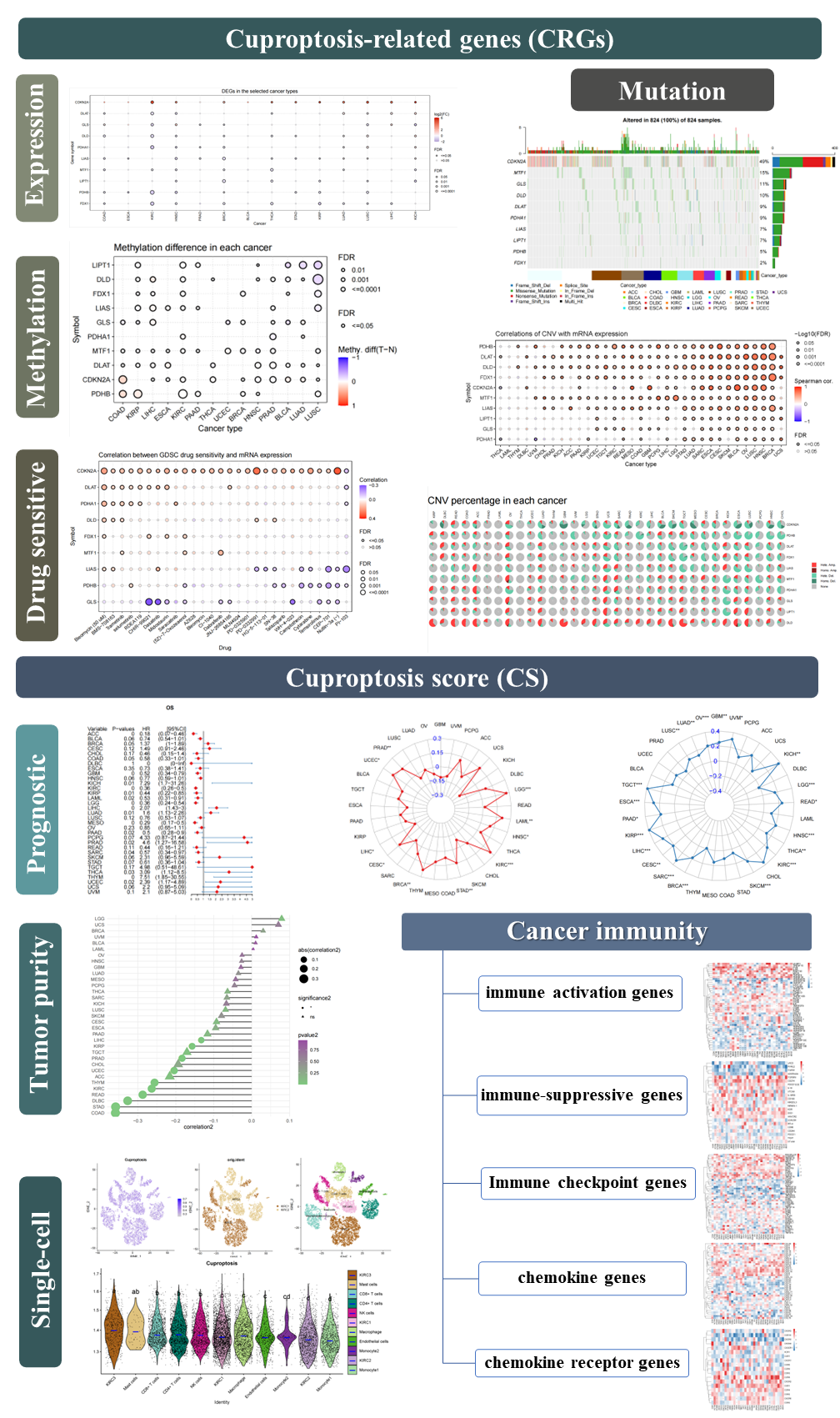


**Supplementary Figure 1.** The flow chart of the study.


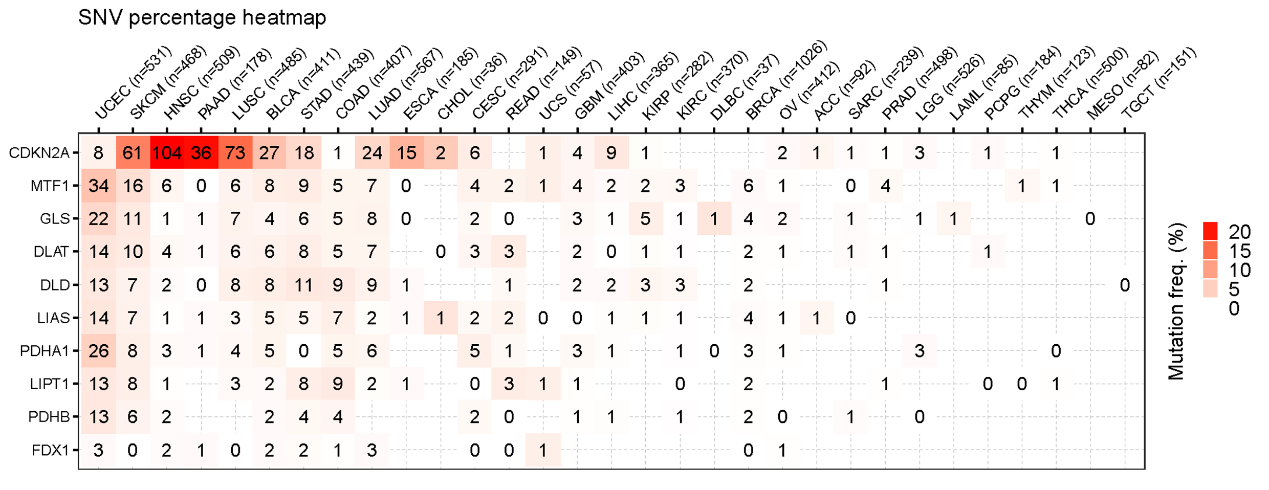
**Supplementary Figure 2. The SNV frequency of CRGs in cancers.** The redder the color, the higher the mutation frequency. Numbers represent the percentage of samples that have the corresponding mutated gene for a given cancer. 0 indicates that there was no mutation in the gene coding region, and no number indicates that there was no mutation in the gene anywhere.


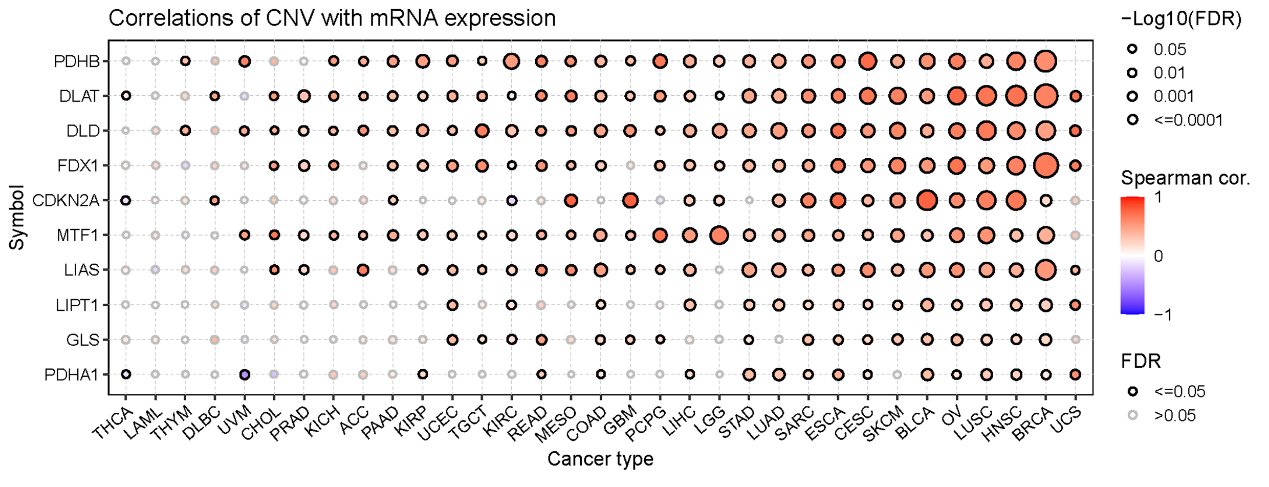
**Supplementary Figure 3. CNV correlation with mRNA expression.** The size of the point represents the statistical significance, where the bigger the dot size, the higher the statistical significance.


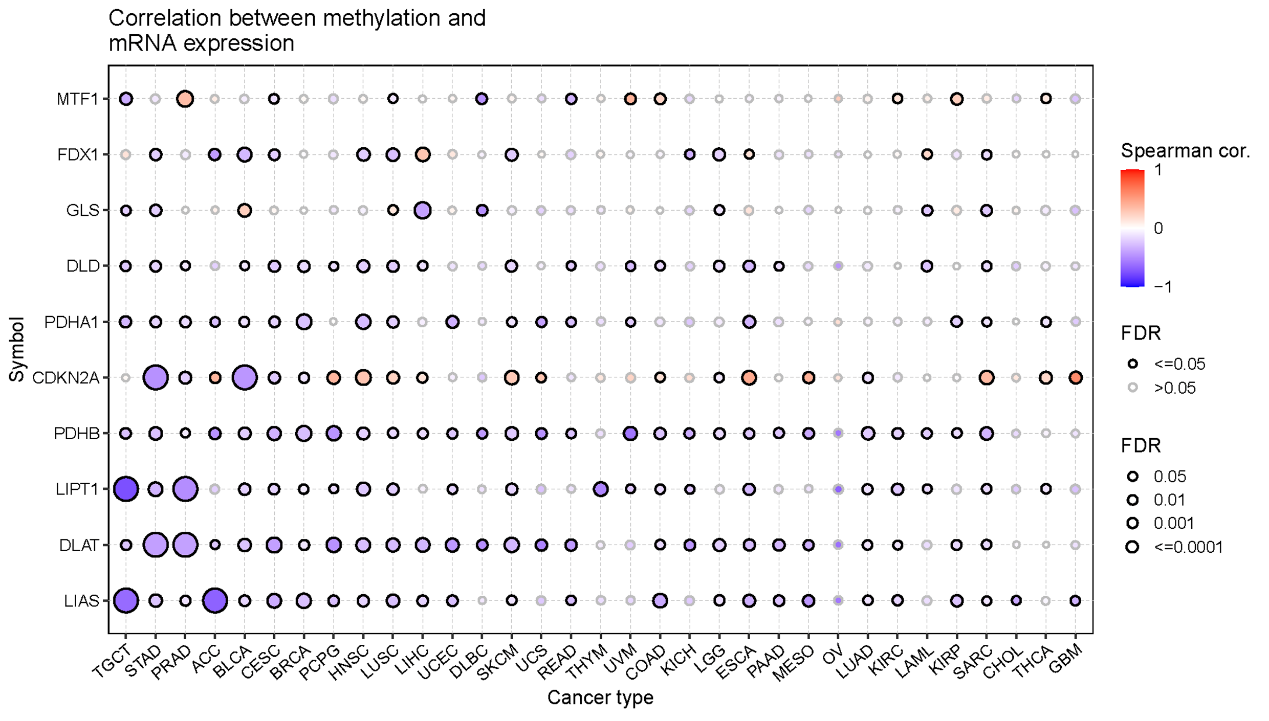
**Supplementary Figure 4. Correlation between methylation and mRNA expression.** The red dots indicate a positive correlation between methylation and mRNA expression, while the blue dots mean the opposite. The bigger the dots, the more significant the correlation.


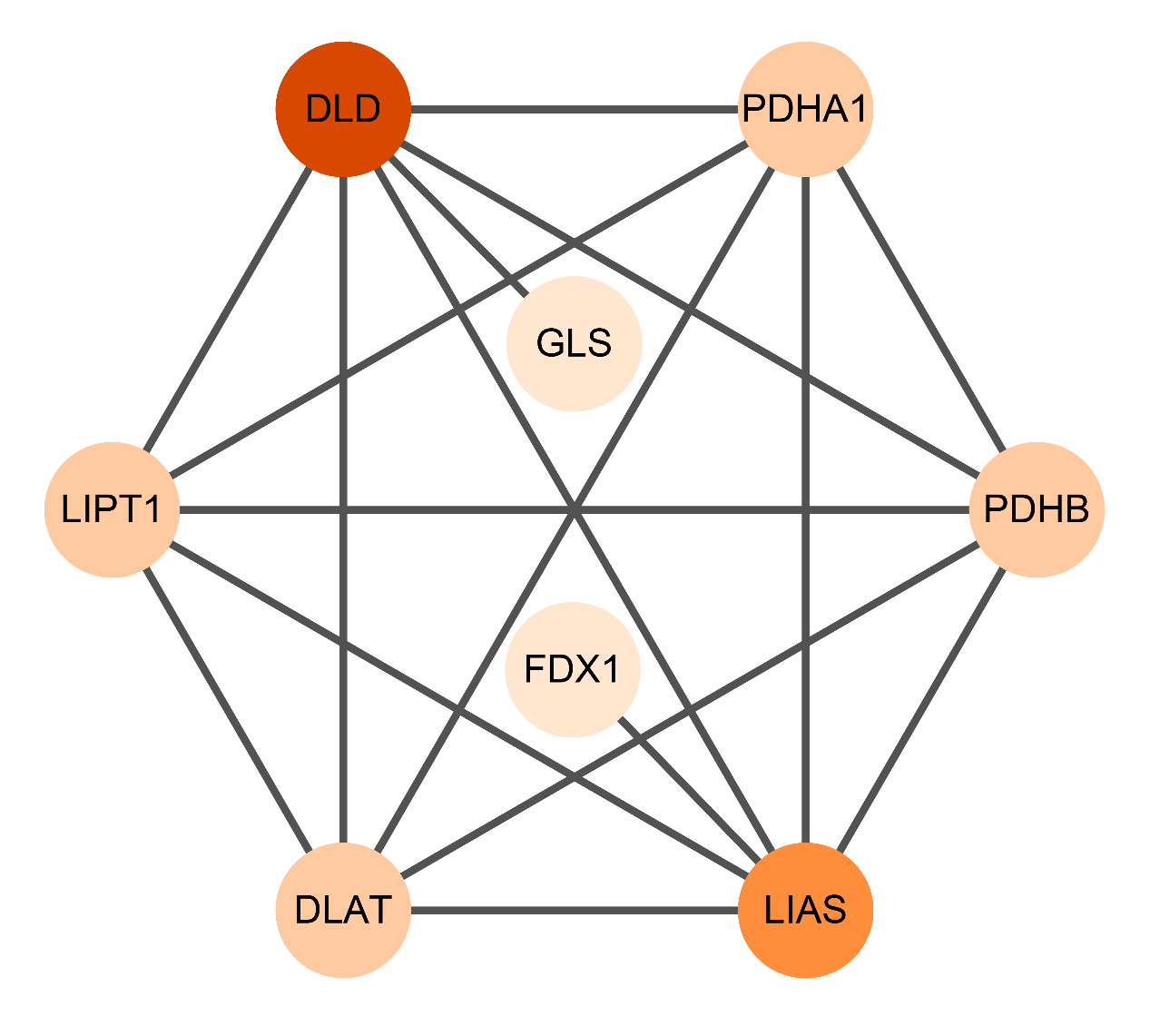


**Supplementary Figure 5.** **Identification of hub genes of CRGs.** Protein network interactions map of CRGs. Proteins are represented as nodes, and the degree of association between proteins is represented as connecting lines.


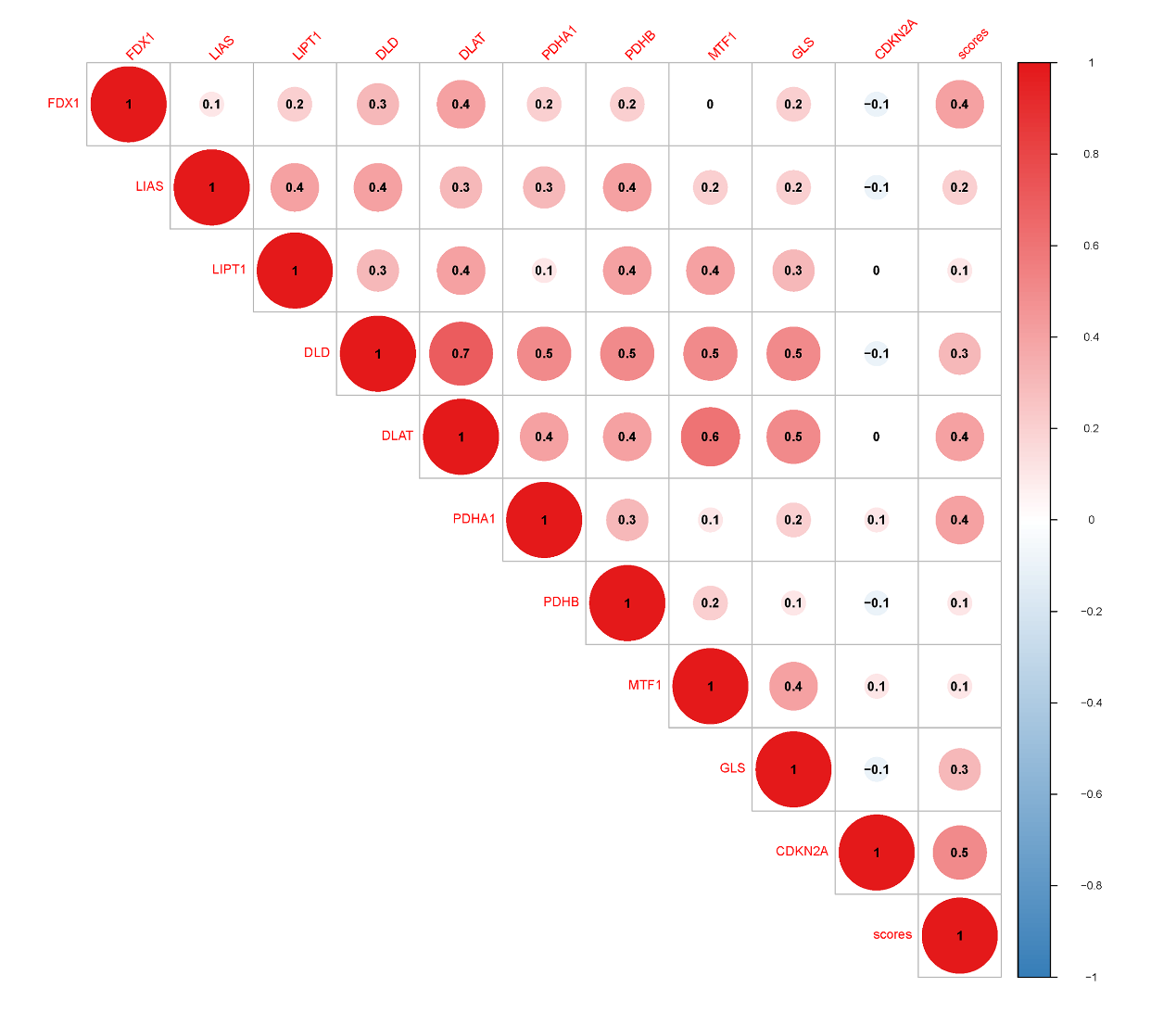
**Supplementary Figure 6. Correlation of cuproptosis multiple genes.** Red represents positive and blue represents negative. The darker the color, the stronger the correlation.


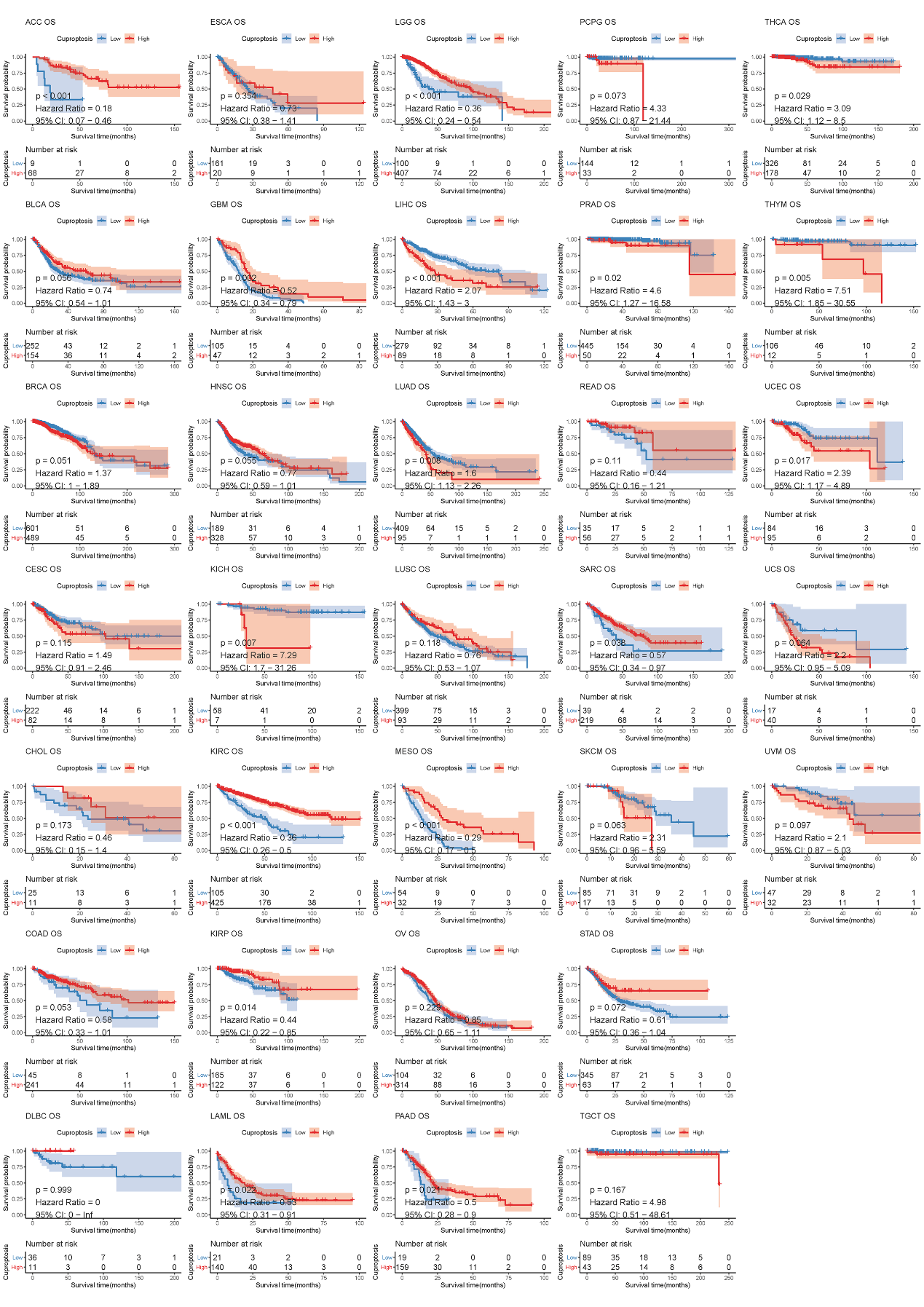
**Supplementary Figure 7.** Kaplan-Meier analysis of the association between CRGs expression and OS.


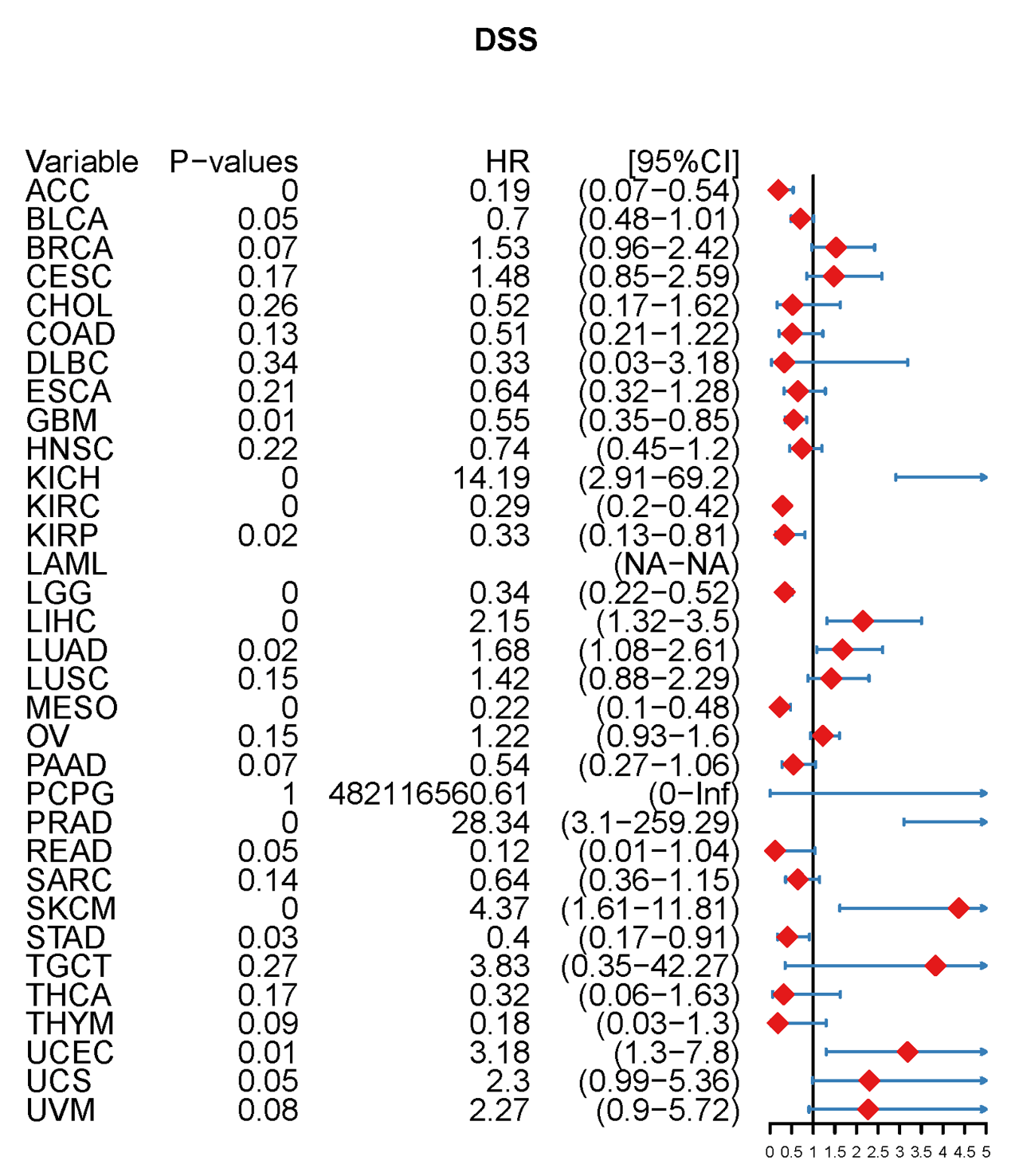
**Supplementary Figure 8.** The results of cuproptosis for disease-specific survival（DSS）in pan-cancer.


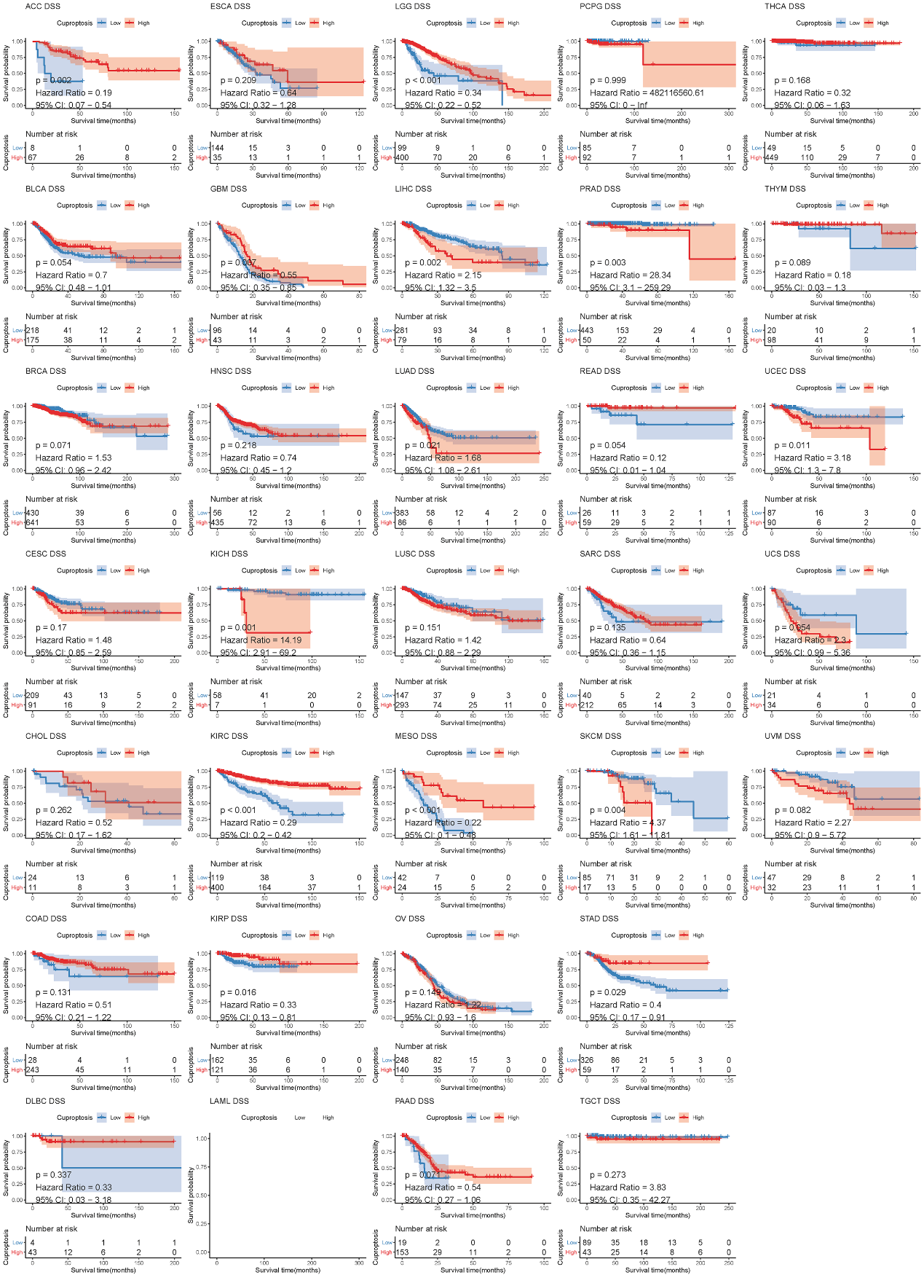
**Supplementary Figure 9.** Kaplan-Meier analysis of the association between CRGs expression and DSS.


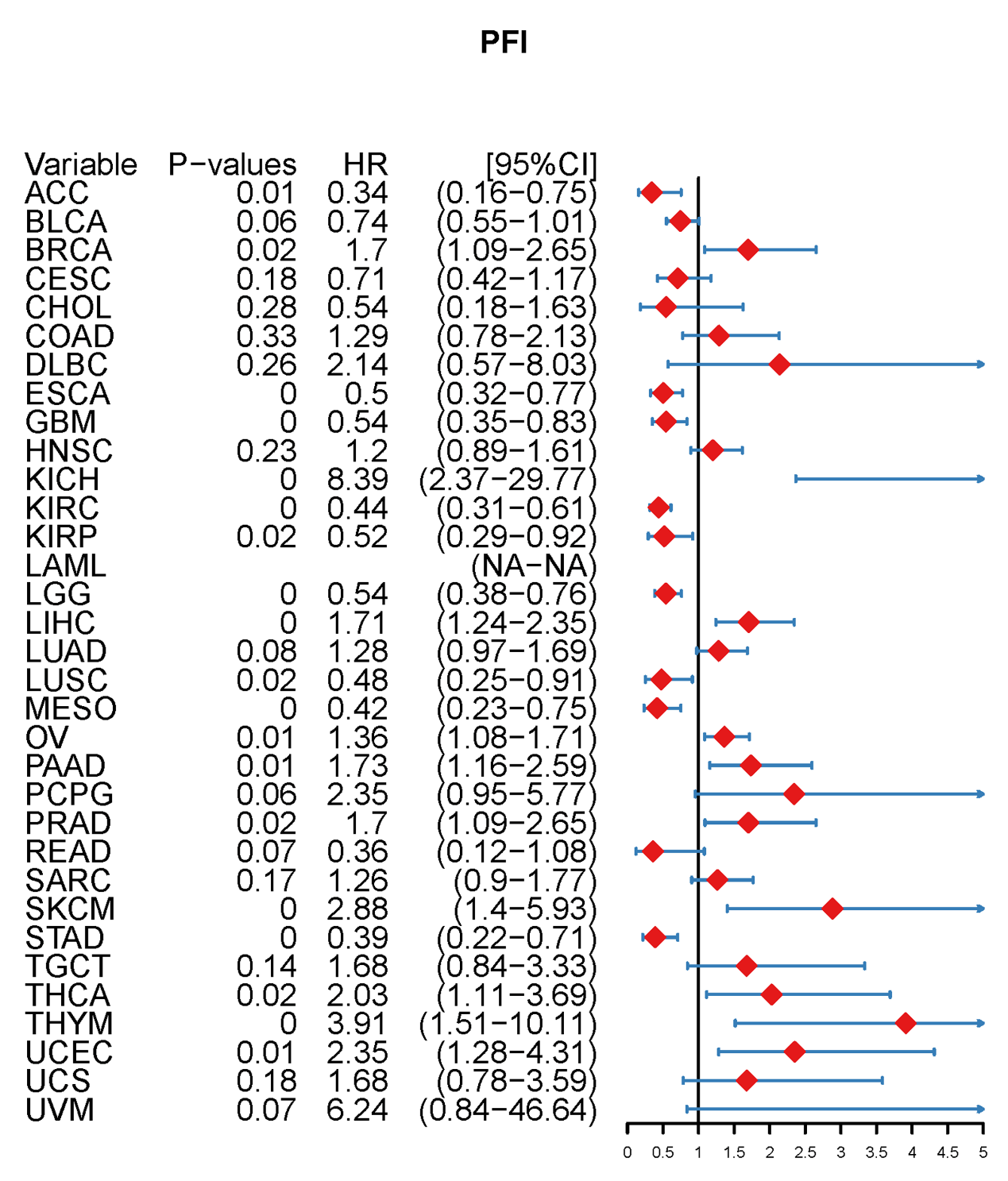
**Supplementary Figure 10.** PFI forest plot for CS.


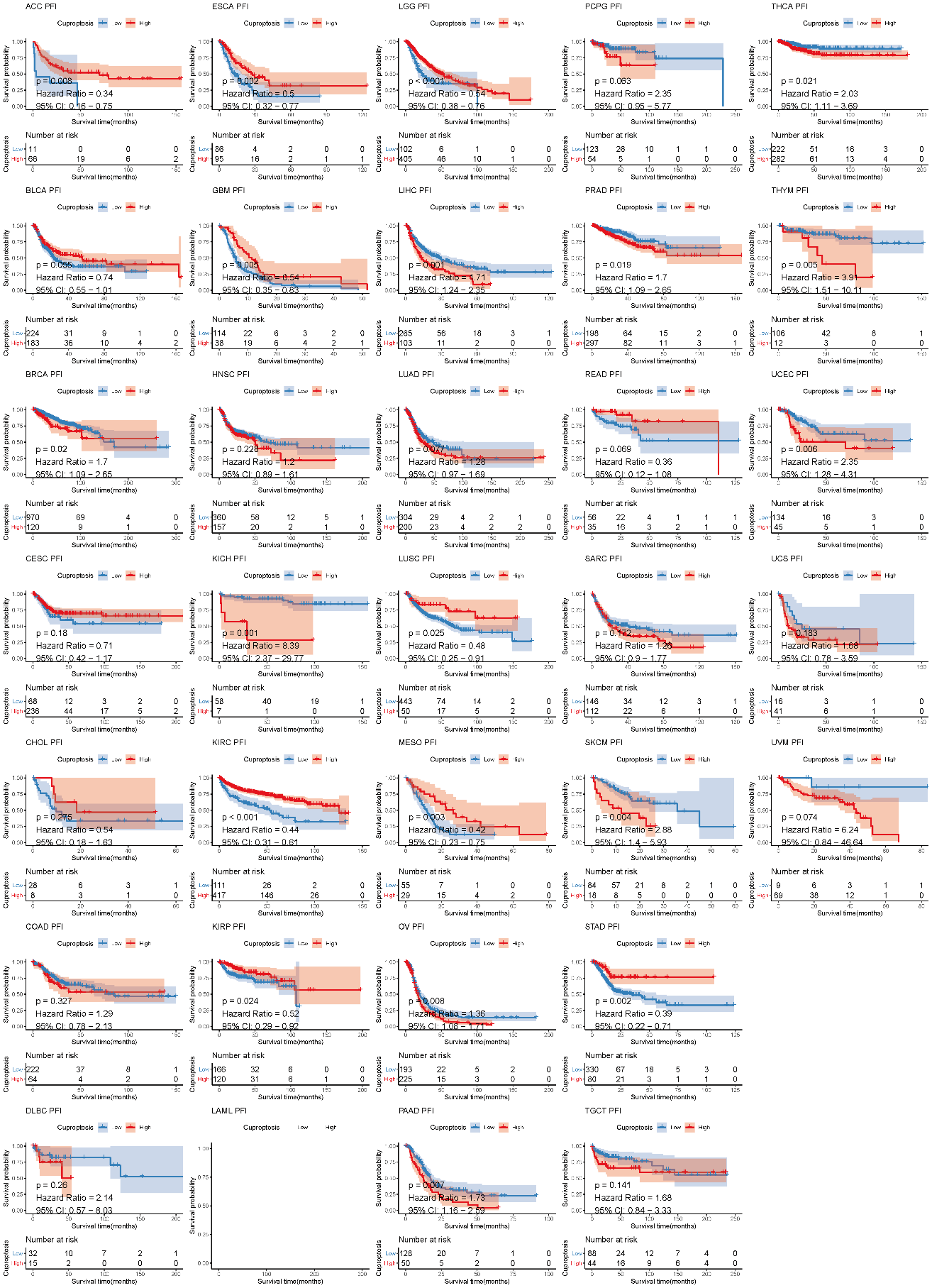


**Supplementary Figure 11.** Kaplan-Meier analysis of the association between CRGs expression and PFI.


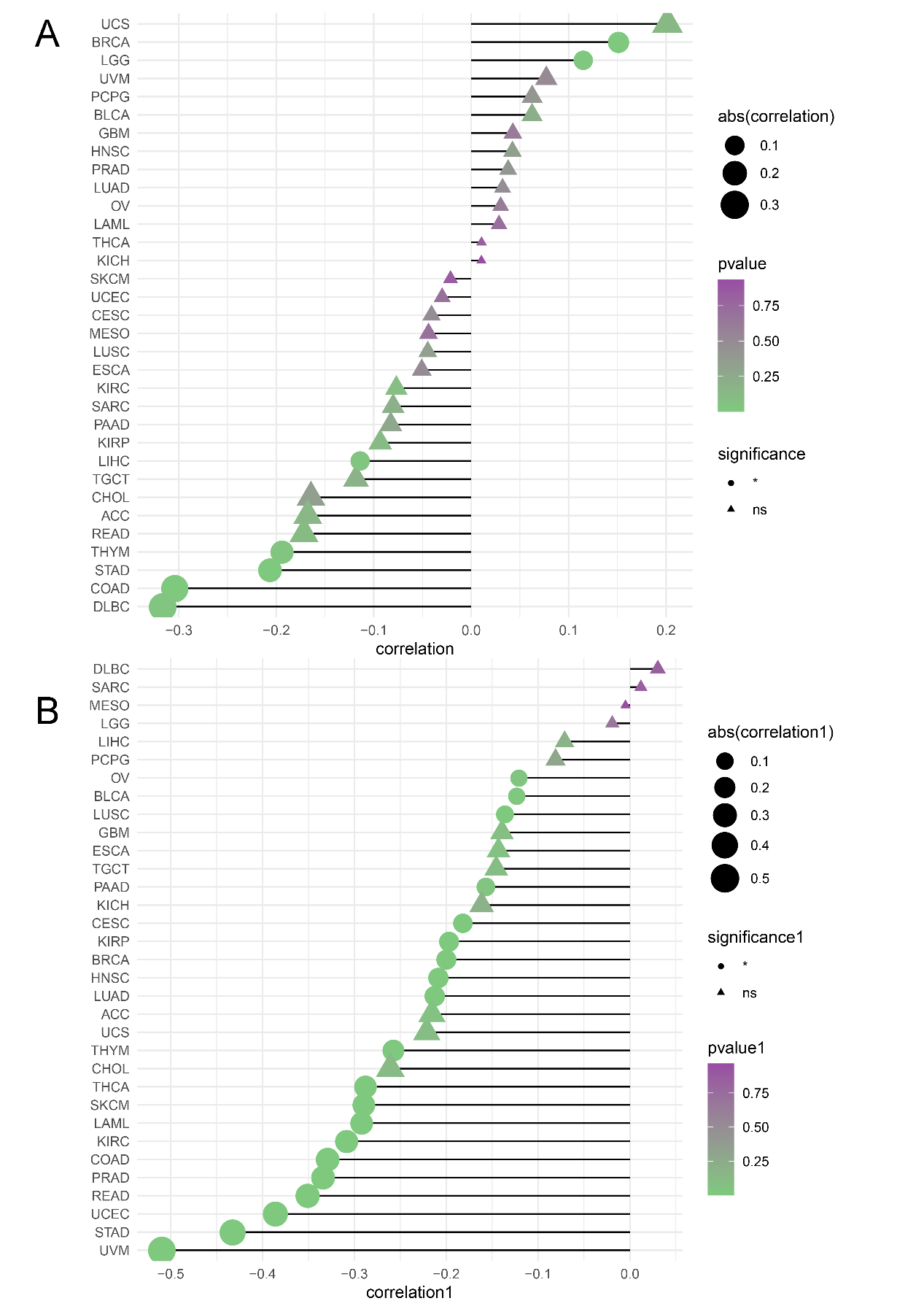


**Supplementary Figure 12. The relationship between CS and immune scores and stroma scores. (A)** Correlation of CS with immune scores. **(B)** Correlation of CS with stroma scores. Triangles indicate no statistical significance and circles indicate statistical significance. The larger the absolute value of the score, the higher the correlation.


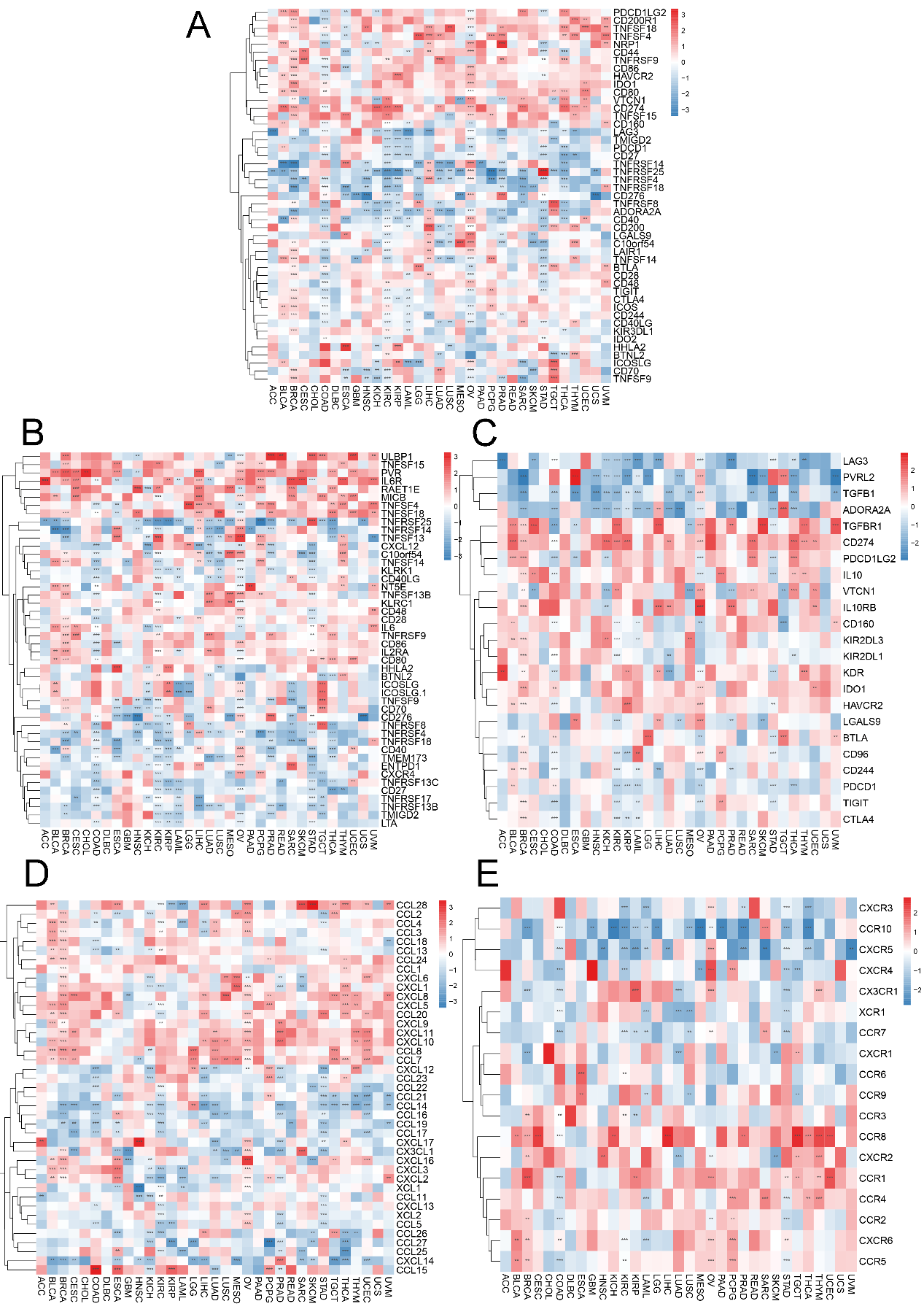


**Supplementary Figure 13. Relationship between CS and immune regulator gene. (A)** Correlation analysis of CS with immune checkpoints. **(B)** Correlation analysis of CS with immune activation genes. **(C)** Correlation analysis of CS with immunosuppressive genes. **(D)** Correlation analysis of CS with chemokines. **(E)** Correlation analysis of CS with chemokine receptors. The redder the color of the box, the higher the degree of correlation between the tumor and the factor of interest, and vice versa. The number of * indicates the degree of correlation.


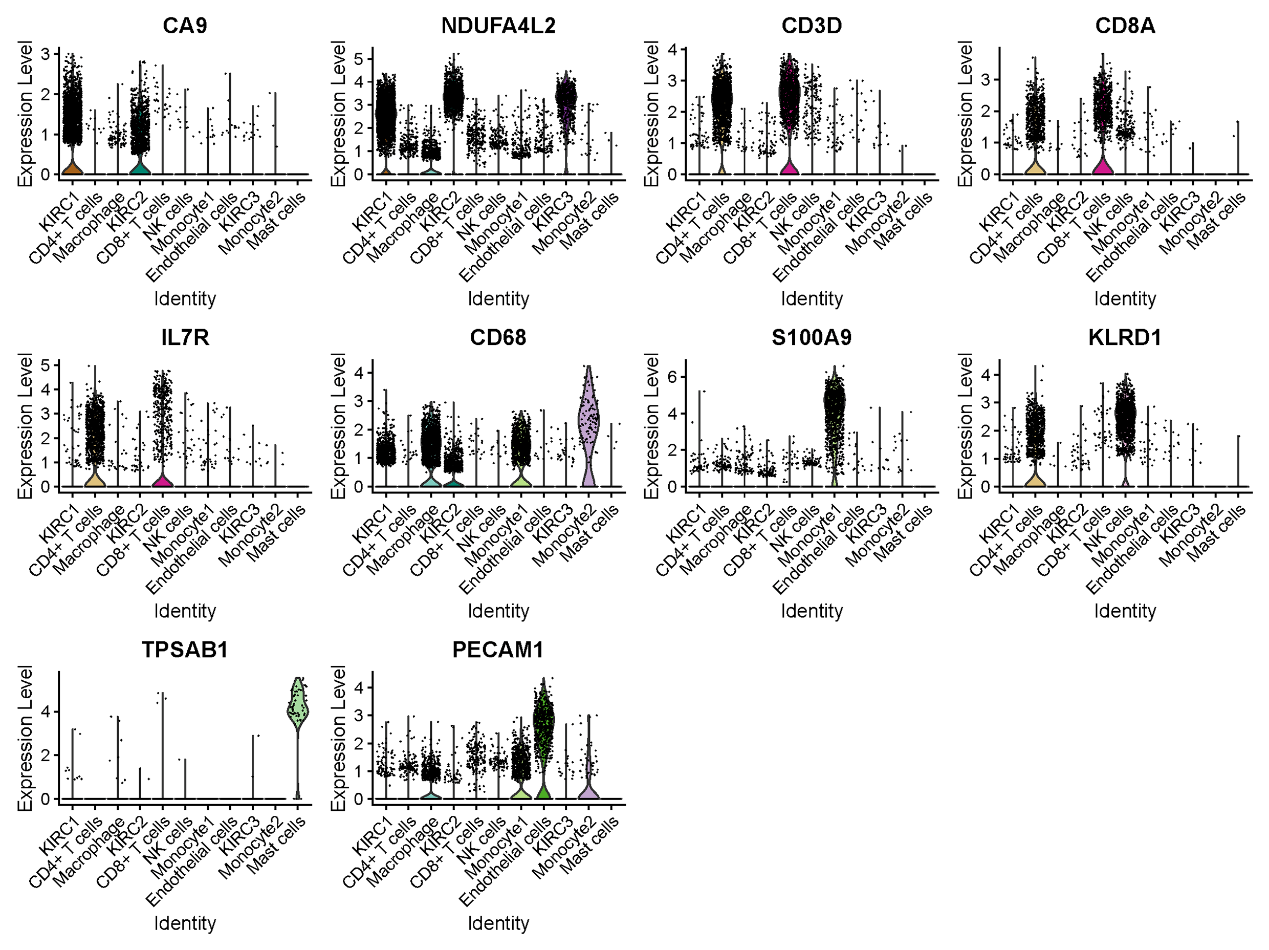
**Supplementary Figure 14.** tSEN plot representation of cuproptosis scores in different cell types.


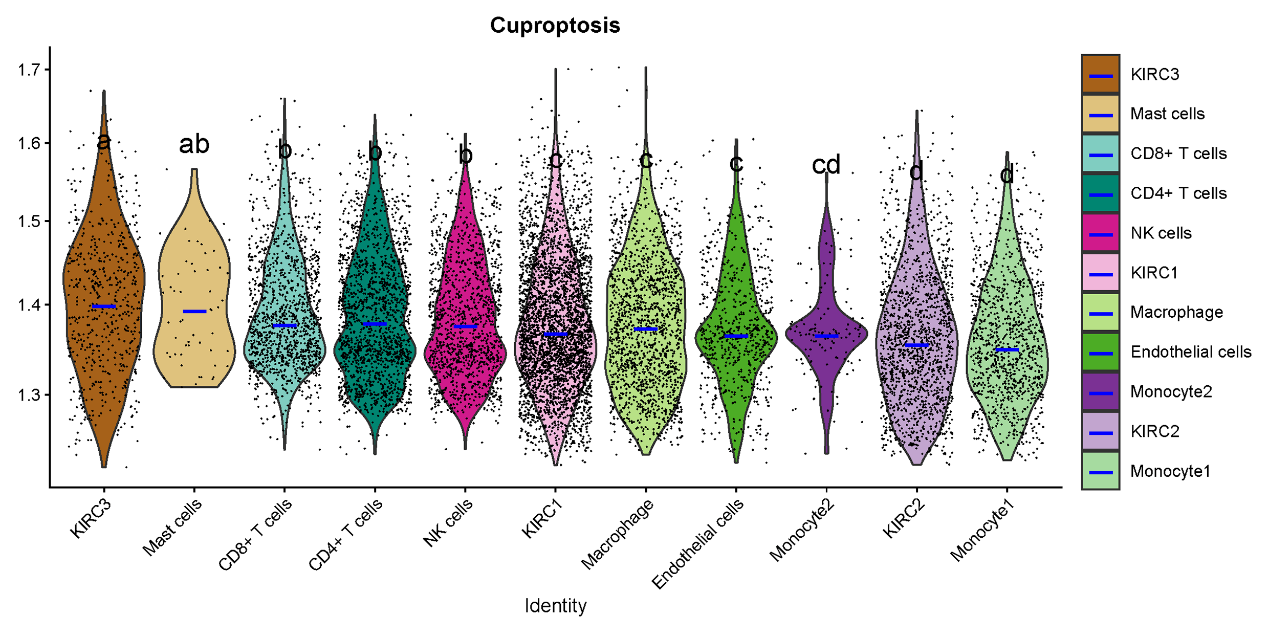
**Supplementary Figure 15. Comparison of CS of different KIRC tumor microenvironments.** The letters at the top indicate whether there is a statistical difference between two comparisons between cells. Different letters indicate that the difference is statistically significant.

**Materials and Methods**

**Single-cell transcriptome sequencing (scRNA-Seq) data analysis**

The preparation and data analysis of scRNA-Seq was performed as previously described [1]. The single-cell sequencing datasets was stored in GEO database (GSE152938). In brief, fresh tumor samples were obtained from the operating room to the laboratory in cold Hank’s balanced salt solution. After the samples were washed and were cut into 2–4 mm pieces. The tissue species were digested for 30 min at 37°C with gentle agitation in a digestion solution in HBSS. Samples were washed, filtered, erythrocytes removed and cell viability assayed before single cell sequencing. Two kidney renal clear cell carcinoma (KIRC) samples were collected from patients undergoing radical nephrectomy. The patients were not receiving any anti-tumor treatment therapy prior to sampling, including chemotherapy, radiotherapy, immunotherapy and Chinese medicine. All samples were sequenced using the Hiseq X10 (Illumina, San Diego, CA) with standard parameters. Preliminary sequencing files (.bcl) were converted to FASTQ files on CellRanger (version 3.0.2). R (version 3.5.2) and Seurat R package (version 3.1.1) were used for Quality Control (QC) and secondary analysis.

**Paraffin-embedded tissue collection**

Paired cancers and paracancerous tissues were derived from 34 lung cancer patients from the Affiliated Cancer Hospital of Guangxi Medical University. All patients were diagnosed with lung cancer and had not received chemotherapy or radiotherapy before tissue collection. Tumor specimens are all derived from surgically excised specimens and the time between isolation and fixation does not exceed 30 minutes. Written informed consent was acquired from all patients. The study was approved by the Ethics and Anthropology Committee of the Affiliated Cancer Hospital of Guangxi Medical University. All experiments and methods were performed in accordance with relevant guidelines and regulations.

**Immunohistochemical staining**

All cancer specimens were immersed in formalin. Additionally, tissues were sliced to a thickness of 5 μm and put on glass slides before staining. By deparaffinizing, rehydrating, and employing 5% bovine serum albumin at 37°C for 30 min, endogenous peroxidase activity was suppressed and blocked. The treated sections were then washed three times with PBS before being incubated with anti-DLD (bioworld BS7282) at 4°C overnight. After that, it must be incubated with sunflower secondary anti-peroxidant for 30 minutes at 37°C. The sections were then developed in diaminobenzidine and microscopic images by light microscopy after washing three times again with PBS.

**Data acquisition and processing**

10 cuproptosis-related genes (FDX1, LIAS, LIPT1, DLD, DLAT, PDHA1, PDHB, MTF1, GLS, and CDKN2A) was obtained from the article: copper induces cell death by targeting lipoylated TCA cycle proteins, published in Science recently[2]. The Genotype-Tissue Expression (GTEx) dataset (V7.0) (https:// commonfund.nih.gov/GTEx/) was used to the analysis of gene expression in healthy people' normal tissues[3]. The Cancer Genome Atlas (TCGA) database preserved more than 10,000 tumor samples clinical and genetic data based on 33 human cancers, including cope number variation (CNV), single nucleotide variation (SNV), mRNA Seq, clinical and methylation data.

**Survival Analysis**

Clinical survival data and mRNA expression were combined using the sample barcode for expression survival analysis. Using the median RSEM value, groups of tumor samples with "high" and "low" gene expression were distinguished. R software package "survival" (https://www.rdocumentation.org/packages/survival) was used to draw Kaplan Meier survival curve analysis of survival difference between high and low expression group[4]. The relationship between the score and overall survival (OS), progression-free survival (PFI) and disease-specific survival (DSS) was explored. The Cox proportional hazards model was built through the R package. Gene data with Kaplan-Meier log-rank test p-values < 0.05 were retained.

**SNV Analysis**

SNV data for 33 cancers were acquired and studied through the TCCG database. Maftools was used to generate the SNV oncoplot, also known as the waterfall plot[5]. The number of mutated samples was divided by the total number of cancer samples to determine the percentage of SNVs in each gene's coding region. Through the R package, SNV data and clinical overall survival data were integrated to evaluate the survival difference between mutant and non-mutated genes

**CNV Analysis**

Using GISTICS 2.0(www.genepattern.org/modules/docs/GISTIC_2.0), raw data were collected from 33 tumors and classified as either homozygous or heterozygous. The percentage of homozygous or heterozygous CNV, as well as the percentages of CNV amplification and deletion for each gene in each cancer, were displayed in the homozygous or heterozygous CNV profile. Next, we used GISTIC-processed CNV data for percentage calculations of CNV subtypes, and only genes with >5% CNV were considered significant. The TCGA barcodes of a sample were used to combine the mRNA expression and CNV data. A Pearson product-moment correlation coefficient and t-distribution were used to identify the relationship between matched mRNA expression and CNV proportion.

**Methylation Analysis**

Methylation data of 14 cancers from corresponding tumor and adjacent normal tissue samples was analyzed. The TCGA barcode of a sample was used to combine the mRNA expression and methylation data. Based on a Pearson product-moment correlation coefficient and t-distribution, the relationship between paired mRNA expression and methylation was investigated. Next, clinical overall survival data and gene methylation data were merged and divided into two groups based on intermediate methylation. Additionally, the Cox coefficient was utilized to calculate the risk of mortality[6]. Hyper-worse was considered as high risk if the Cox coefficient was less than 0 and the survival prognosis for the hypermethylated group was bad.

**Evaluation of Cuproptosis score**

The CRGs set was downloaded from the GSEA database to quantify the expression levels of CRGs for each cancer. Next, we calculated the Cuproptosis score based on the single-sample gene-set enrichment analysis (ssGSEA)[7].

**Correlation analysis between** **Cuproptosis and tumor immune invasion**

MRNA sample sequences from TCCG were evaluated. Moroever, immune cell integration (gepia.cancer-pku.cn) was conducted using the TIMER (timer.cistrome.org) and GEPIA databases[8, 9]. Using Immune Cell Infiltration Analysis TIMER2, we investigated the association of CRGs with tumor stromal cells and tumor-infiltrating immune cells.

**Pathway exploration for** **Cuproptosis in pan-cancer**

The functions and pathways of Cuproptosis in pan-cancer were further explored through different databases. Based on the CancerSEA database ([home (hrbmu.edu.cn)](http://biocc.hrbmu.edu.cn/CancerSEA/home.jsp)), the correlation between circadian rhythm-related genes and functional status in different tumors was analyzed[10]. In addition, the correlations between the Cuproptosis and functional states in various cancers was analyze by the CancerSEA database.

**TIDE analysis**

Potential ICB response was predicted based on the Tumor Immune Dysfunction and Exclusion (TIDE) algorithm[11]. TIDE can be used to assess two tumor immune escape pathways. The likelihood that tumor cells will elude detection and attack by the body's immune system through a variety of ways increases with increasing TIDE score.

Reference

1. Su, C., et al., *Single-Cell RNA Sequencing in Multiple Pathologic Types of Renal Cell Carcinoma Revealed Novel Potential Tumor-Specific Markers.* Front Oncol, 2021. **11**: p. 719564.

2. Tsvetkov, P., et al., *Copper induces cell death by targeting lipoylated TCA cycle proteins.* Science, 2022. **375**(6586): p. 1254-1261.

3. *Human genomics. The Genotype-Tissue Expression (GTEx) pilot analysis: multitissue gene regulation in humans.* Science, 2015. **348**(6235): p. 648-60.

4. Sha, S., et al., *Prognostic analysis of cuproptosis-related gene in triple-negative breast cancer.* Front Immunol, 2022. **13**: p. 922780.

5. Mayakonda, A., et al., *Maftools: efficient and comprehensive analysis of somatic variants in cancer.* Genome Res, 2018. **28**(11): p. 1747-1756.

6. Tibshirani, R., *The lasso method for variable selection in the Cox model.* Stat Med, 1997. **16**(4): p. 385-95.

7. Chen, M., et al., *A New Ferroptosis-Related lncRNA Signature Predicts the Prognosis of Bladder Cancer Patients.* Front Cell Dev Biol, 2021. **9**: p. 699804.

8. Li, T., et al., *TIMER: A Web Server for Comprehensive Analysis of Tumor-Infiltrating Immune Cells.* Cancer Res, 2017. **77**(21): p. e108-e110.

9. Tang, Z., et al., *GEPIA: a web server for cancer and normal gene expression profiling and interactive analyses.* Nucleic Acids Res, 2017. **45**(W1): p. W98-w102.

10. Yuan, H., et al., *CancerSEA: a cancer single-cell state atlas.* Nucleic Acids Res, 2019. **47**(D1): p. D900-d908.

11. Jiang, P., et al., *Signatures of T cell dysfunction and exclusion predict cancer immunotherapy response.* Nat Med, 2018. **24**(10): p. 1550-1558.
